# Analysis of rare genetic variants in *All of Us* cohort patients with common variable immunodeficiency

**DOI:** 10.3389/fgene.2024.1409754

**Published:** 2024-10-02

**Authors:** Troy von Beck, Meera Patel, Niraj C. Patel, Joshy Jacob

**Affiliations:** ^1^ Department of Pediatrics, Division of Medical Genetics, Duke University, Durham, NC, United States; ^2^ Department of Pediatrics, Division of Allergy and Immunology, Duke University, Durham, NC, United States; ^3^ Emory Vaccine Center, Emory National Primate Research Center, Emory University, Atlanta, GA, United States

**Keywords:** common variable immunodeficiency, hypogammaglobulinemia of undetermined significance, *All of Us*, pathogenicity prediction, genetics

## Abstract

Common variable immunodeficiency (CVID) is a group of genetic disorders involving more than a dozen genetic loci and characterized by a deficiency in specific antibody isotypes leading to poor immune responses and recurrent infection. CVID affects approximately 1 in 10,000 to 1 in 50,000 people worldwide with substantial heterogeneity in disease severity, including asymptomatic individuals designated as hypogammaglobulinemia of undetermined significance (HGUS). As expected of humoral immunodeficiency, the molecular causes of CVID primarily affect the maturation, activation, or survival of B cells and plasma cells. In this retrospective analysis, we defined a cohort of 21 patients with a primary CVID or HGUS diagnosis in the v7 release of the *All of Us* Research Program database and performed gene annotation and variant effect prediction. Our analysis identified both known disease-causing variants and rare genetic variants overlapping with other immunodeficiency syndromes.

## 1 Introduction

Common variable immunodeficiency (CVID) is a term used to classify a collection of related genetic disorders affecting roughly 1 in 10,000 to 1 in 50,000 globally and characterized by the absence or severe deficiency for IgG, IgA, and occasionally IgM antibody isotypes (Salzer et al., 2012). Currently, 14 monogenic causes of CVID have been identified and at least 60 genes are suggested as putative risk alleles or disease modifiers (Hamosh et al., 2005; de Valles-Ibanez et al., 2018). The great breadth of genetic loci implicated in CVID pathogenesis also underlies the significant heterogeneity of disease presentations in affected individuals. Most individuals are diagnosed with CVID during adulthood in the 3^rd^ or 4^th^ decade of life, suggesting a combination of genetic risk factors, immunosenescence, and environmental exposures dictate disease progression (Salzer et al., 2012). However, the condition may also occur in children and adolescents, but is more difficult to distinguish from naturally low immunoglobulin titers in the developing immune system (Szczawinska-Poplonyk et al., 2022). Due to the deficiency of immunoglobulin, CVID patients typically present with recurrent bacterial and viral infections as well as inadequate responses to vaccination (Orange et al., 2012). The altered immune function also places individuals at a greater risk of autoimmunity and lymphoid malignancy (Salzer et al., 2012). Some individuals may present asymptomatically with low immunoglobulin titers and be classified under the related condition, hypogammaglobulinemia of undetermined significance (HGUS). Overtime, many patients with HGUS will progress to CVID as the two conditions likely share the same genetic determinants (Ameratunga et al., 2013).

Regarding the molecular pathogenesis of CVID, most risk alleles affect the maturation, activation, or survival of B cells and antibody secreting plasma cells. These defects can be either B cell intrinsic, as in the case of complement receptor or NF-κB variants, or extrinsic, as in the case of LRBA variants affecting CTLA-4 expression on T cells. Common variants affecting B cell survival include variants in the BAFF receptor (BAFF-R) and TACI which impair the ability of B cells and plasma cells to receive pro-survival signals from BAFF ligand and APRIL (Martinez-Gallo et al., 2013; Block et al., 2023). Similarly, variants affecting maturation or activation include polymorphisms in core transcription factors such as NF-κB or Ikaros (Eskandarian et al., 2019; Tuijnenburg et al., 2018). As only about 25%–30% of CVID patients have an identifiable monogenic origin (Abolhassani et al., 2020; Rojas-Restrepo et al., 2021), it is likely that the disease is typically the result of multiple minor genetic insufficiencies leading to a profound collapse in antibody production (de Valles-Ibanez et al., 2018). Therefore, further investigation is required to identify all the key risk loci of CVID, both for better patient stratification and the design of improved treatments and curative therapies.

The *All of Us* Research Program (https://www.researchallofus.org/data/workbench/) funded by the National Institutes of Health (NIH) provides unprecedented access to patient genomic data with paired electronic health records (EHR) (All of Us Research Hub, 2024). The *All of Us* program has a stated goal of enrolling at least 1 million participants from the United States of America with a diverse representation. The integration of whole genome sequencing, EHR data, health questionnaires, and physical measurements aims to create a powerful cohort to advance biomedical research which can further account for individual differences in environment, lifestyle, socioeconomic factors, and biological characteristics (Denny et al., 2019). Using this database, we curated a cohort of 21 individuals with an apparent primary chronic CVID or HGUS diagnosis from the 245,388 whole genome sequences (WGS) available in the Controlled Tier Dataset version 7. We then performed gene annotation and filtering to identify highly probable damaging variants using computational measures of allele frequency, evolutionary conservation, and deleteriousness. Our results identify both well-characterized risk alleles and novel genetic variants located at putative CVID loci.

## 2 Methods

### 2.1 Cohort curation in the *All of Us* v7 dataset

The *All of Us* cohort builder was used with version 7 of the dataset to screen 245,388 individuals with available whole genome sequence data (All of Us Research Hub, 2024). Patients were included in the analysis if they had a chronic diagnosis of CVID or HGUS, defined here as at least two mentions of either CVID or hypogammaglobulinemia at least 1 year apart in their electronic health records. As a note, some individuals with HGUS had a singular mention of CVID in their EHR, which does not meet our criteria for defining chronic CVID (2 mentions 1-year apart), therefore these individuals were grouped with the HGUS patients. To exclude patients with non-genetic forms of CVID/HGUS, we filtered out patients with the terms “malignant neoplastic disease”, “human immunodeficiency virus”, or “transplant present” (condition concept covering all organ/tissue transplant recipients) mentioned before a CVID/HGUS diagnosis. We further excluded patients prescribed any of the following drugs prior to a mention of CVID/HGUS: Belimumab, Rituximab, Obinutuzumab, Ofatumumab, Alemtuzumab, Methotrexate, Hydroxychloroquine, Baricitinib, Tofacitinib, Upadacitinib, Sulfasalazine, Chlorambucil, Cyclophosphamide, Melphalan, or Mycophenolate. Finally, to exclude individuals with CVID/HGUS due to immune senescence, we excluded individuals >50 years of age or who were deceased at the time of analysis. This yielded a participant count of 22 individuals with chronic CVID and 20 individuals with chronic HGUS whose EHRs were then evaluated by a clinical immunologist for substantiating diagnostic evidence of recurrent infections, low immunoglobulin titers, and replacement immunoglobulin therapy. Based on the manual review of patient EHRs, 14 of the 22 CVID and 7 of the 20 HGUS patients met the inclusion criteria and were selected for further bioinformatics analysis. Publication of individual genetic variants, demographic factors, and the exact patient count in this small-cohort study was approved by the *All of Us* Resource Access Board under a data and statistics policy exception request.

### 2.2 Stratification of genetic variants

Whole genome sequence data was first annotated using SnpEff with the hg38 reference genome (GRCh38.99) (Cingolani et al., 2012a). SnpSift was then used to identify potential functional variants annotated with an “impact” score of “high” or “moderate” (Cingolani et al., 2012b). SnpSift was used to annotate the PhastCons and gnomAD allele frequencies and CADD v1.6 scores using the phastCons100way (https://hgdownload.cse.ucsc.edu/goldenPath/hg38/phastCons100way/), gnomAD exomes 2.1.1 hg38 lift over (https://gnomad.broadinstitute.org/), and CADD v1.6 (https://cadd.gs.washington.edu/download) for gnomAD genomes 3.0.0 files respectively (Siepel et al., 2005; Rentzsch et al., 2021; Karczewski et al., 2020). SnpSift was then used to filter out variants with a PhastCons score < 0.9, an allele frequency > 0.01, a CADD Phred score below 20, or a Phred quality score below 20. For homozygous variation, alleles were further excluded if they were observed in more than two individuals.

## 3 Results

To begin the analysis, 14 individuals with chronic CVID and 7 individuals with chronic HGUS were identified in the *All of Us* v7 patient cohort. These individuals were selected based on mentions of either CVID or HGUS more than 1 year apart in their medical records along with a clinical history of recurrent infections, low immunoglobulin laboratory values, replacement immunoglobulin therapy, and the absence of confounding non-genetic factors such as chemotherapy or immunosuppression. To explore potential genetic causes of antibody deficiency in this population, WGS data was extracted then annotated and filtered using the SnpEff and SnpSift packages (Cingolani et al., 2012a; Cingolani et al., 2012b) to isolate protein coding non-synonymous variation. As a first step, protein coding variants affecting Online Mendelian Inheritance in Man (OMIM) (Hamosh et al., 2005) defined CVID loci were screened for prior mention in CVID literature (Table 1). Several variants were identified in both patient populations with varying levels of support for their pathogenic significance. The most substantiated are the C104R variant in TACI (*TNFRSF13B*) and P21R variant in the BAFF receptor (*TNFRSF13C*). Both variants reduce signaling through their respective receptor but should be considered as CVID risk factors due to their relatively high population frequency and incomplete penetrance in heterozygous individuals (Block et al., 2023; Lee et al., 2010). Recently, the M467V variant in *LRBA* was reported to occur heterozygous in a large cohort of CVID individuals, but without functional characterization (Cunningham-Rundles et al., 2023). The CVID patient bearing the M467V LRBA variant also carried the N815S LRBA variant described in Table 2, possibly indicating a compound heterozygous status. One patient is also heterozygous for the *IRF2BP2* P118S variant, although this variant lacks substantial characterization from either functional or inheritance perspectives (Schröder et al., 2019).

**TABLE 1:** Previously described CVID risk alleles observed in this cohort.

Gene	cDNA	AA change	Allele count	gnomAD_AF	CADD Phred	PhastCons	Functional effect
*IRF2BP2*	c.352C>T	P118S	1	0.022026	14.6	0.991	
*LRBA*	c.1399A>G	M467V	1	0.002123	22.5	1.000	
*TNFRSF13B*	c.310T>C	C104R	1	0.003500	25.8	1.000	Impaired BAFF and APRIL binding (Martinez-Gallo et al., 2013)
*TNFRSF13C*	c.62C>G	P21R	1	0.060653	8.226	N/A	Reduced BAFF-R multimerization (Lee et al., 2010)
*TNFRSF13B*	c.310T>C	C104R	2	0.003500	25.8	1.000	Impaired BAFF and APRIL binding (Martinez-Gallo et al., 2013)

Variants from CVID and HGUS patients are shown in yellow and blue respectively.

**TABLE 2:** Rare functional variants observed at putative CVID loci.

Gene	cDNA	AA change	Allele count	gnomAD_AF	CADD Phred	PhastCons	Functional effect
*LRBA*	c.8233C>T	L2745F	1	0.000326	28.5	1	
*LRBA*	c.2444A>G	N815S	1	0.002211	23.8	1	Possible relationship to Hirschsprung’s disease (Sribudiani et al., 2018)
*BLK*	c.713G>A	R238Q	2	0.003074	27.1	1	SLE association, Lacks kinase activity *in vitro* (Jiang et al., 2019)
*BLK*	c.1366C>T	R456C	1	0.000295	20.1	N/A	Possible relationship to mature onset diabetes of the young (MODY) (Taha et al., 2023)
*DOCK8*	c.3704C>T	A1235V	1	0.000008	N/A	1	
*RAG1*	c.906C>A	D302E	1	0.006173	22.6	0.991	
*CLEC16A*	c.2945G>A	S982N	1	0.005132	25.3	1	
*TCF3*	c.302A>G	K101R	1	0.009470	23.9	0.952	
*LONP1*	c.1612C>T	R538C	1	0.000334	31	1	
*LONP1*	c.1309G>A	V437I	1	0.003074	24.7	1	
*DOCK8*	c.3460C>T	R1154C	1	0.002862	28.6	1	Possible relationship to Hyper-IgE recurrent infection syndrome 2 (HIES2) (Dansu, 2019)
*UNC93B1*	c.1453G>A	V485M	1	0.004220	26.2	1	
*UNC93B1*	c.626C>T	P209L	1	0.003876	23.1	1	
*UNC93B1*	c.385C>A	L129I	1	0.003806	25.6	1	

Variants from CVID and HGUS patients are shown in yellow and blue respectively. Allele frequencies are rounded to the nearest millionth place.

Following this preliminary screening, the scope of analysis was expanded to include 61 genes implicated in antibody deficiency disorders, including the 14 OMIM defined genes (Supplementary Table S1). As this rapidly expands the number of potential variants, a screening protocol based on evolutionary conservation (phastCons100way score), population frequency (gnomAD allele frequency), and predicted deleteriousness (CADD score) was employed to isolate significant novel variants. For this screening, variants with a WGS Phred quality score greater 20, a phastCons100way (Siepel et al., 2005) score of greater than 0.9, a Genome Aggregation Database (gnomAD) exomes (Karczewski et al., 2020) allele frequency below 1%, and a Combined Annotation Dependent Depletion (CADD) PHRED (Rentzsch et al., 2021) score greater than 20 were considered significant (Table 2). As some variants lacked annotation in one of the 3 databases, we included those with any 2 of the 3 annotations. Notably, none of the variants observed in Table 2 were found in homozygosity for any patient. The *BLK* R238Q was observed at a higher-than-expected frequency in this cohort. Notably, this variant occurs at a residue conserved across Src-kinases and is documented to ablate BLK kinase activity in transfection experiments (Jiang et al., 2019).

Several other significant variants with prior characterization were identified in single individuals. Some of these such as the *LRBA* N815S, *BLK* R456C, and *DOCK8* R1154C are only significant because of their observed occurrences in the context of other genetic diseases (Sribudiani et al., 2018; Taha et al., 2023; Dansu, 2019). For clarification, variants in the *UNC93B1* allele were limited to a single patient.

Finally, individuals were assessed across all genetic loci for the presence of homozygous or male hemizygous stop-gain, start-loss, or frameshift variants (Table 3). As we did not perform phasing of the dataset to distinguish the parentally inherited copies of each chromosome, we were unable to evaluate the contribution of compound heterozygous variants in this analysis. In addition to the previous filtering constraints of conservation, allele frequency, and predicted deleteriousness, we further excluded variants observed in more than 2 individuals in this cohort. The two remaining homozygous variants occurred in *MUC4* and *SPAG11A*, both genes with no known involvement in immunity.

**TABLE 3:** Rare homozygous loss of function variants identified in this cohort.

Gene	cDNA	AA change	gnomAD_AF	CADD Phred	PhastCons	Gene functions in immunity	Zygo
*MUC4*	c.4259_4260insTG	H1421fs	0.000266	21.7	N/A		1 homo
*SPAG11A*	c.281dupT	C95fs	0.000035	29.6	0.997		1 homo

Variants from CVID and HGUS patients are shown in yellow and blue respectively. Allele frequencies are rounded to the nearest millionth place.

## 4 Discussion

In the present study, we analyzed the WGS of 21 individuals presenting with CVID or the related HGUS syndrome. Quantifying well-known risk alleles, we identify damaging TACI and BAFF receptor variants in 4/21 individuals, a rate which agrees with earlier CVID studies (Block et al., 2023; Romberg et al., 2013). However, CVID is rarely monogenic and likely encompasses a spectrum of antibody insufficiency syndromes based on an accumulation of multiple risk alleles.

This study further increases the awareness of potential CVID risk alleles outside of the OMIM defined loci using strict filtering criteria for probably deleterious variants. While most of these are uncharacterized, there is a correlation between the *BLK* R238Q LOF variant and CVID in this cohort, which would support its definition as a risk allele in future studies. We also note a variant in *DOCK8* shared between one HGUS patient in this cohort and a HIES2 patient in a separate cohort (Dansu, 2019). Immunodeficiency is a common trait in HIES2 patients, and it is therefore interesting to consider that partially functional *DOCK8* variants may generate a spectrum of disorders from HGUS to HIES2 with antibody deficiency as the primary symptom. Notably, many of the genes described here are not known to cause CVID with mono allelic inheritance of pathogenic variants such as LRBA and DOCK8. While the described variants could possess uncharacterized dominant negative activity, we speculate a more likely explanation is the presence of compounding effects with other variants including non-coding variants and specific environmental exposures that predispose to CVID. Future investigations will be necessary to experimentally determine the functional consequences of each of the above-described variants and their population frequency in other cohorts.

While this study has identified several novel gene variants with potential implications for primary antibody deficiency, it is also restricted by several limitations. First, the analysis examined a relatively small number of patients, owing to the low frequency of CVID among the general population. We note that of the 42 patients initially identified based on mentions of CVID or HGUS, half of them were excluded from analysis as they lacked appropriate laboratory or clinical data to further substantiate a CVID or HGUS diagnosis despite a history of recurrent infections. We speculate that many of these patients have had substantiating laboratory testing for immunoglobulin, but that this data is siloed in other EHR systems not integrated in the *All of Us* patient dataset. Future releases of the dataset may therefore expand this population as more EHR systems become integrated with *All of Us*. Second, the analysis presented here only included protein coding variants, which likely misses many significant variants affecting gene splicing as well as mRNA transcription and translation. Third, compound heterozygous variants were not evaluated due to an inability to discriminate variants which occurred *in-cis* versus *in-trans*. Lastly, the variants identified here are dependent on the selection of databases for allele frequency, conservation, and predicted deleteriousness scores. Performing this screening again with other resources for each of the above metrics would certainly generate a distinct list of variants, although with a great deal of expected overlap.

Ultimately our understanding of CVID and our ability to stratify the patient population and provide personalized therapeutic regimens is limited by the incomplete characterization of the CVID genetic landscape. For patients without an obvious loss-of-function variant or without a variant credited by one of the large cohort genome wide association studies, there can be only symptomatic management. Modern bioinformatics tools such as those utilized here provide an alternative means to identify high impact variants in patients, accelerating their discovery and driving informed therapeutic decisions.

## Data Availability

The original contributions presented in the study are included in the article/Supplementary Material, further inquiries can be directed to the corresponding author.
