# Supplementary material for: Analysis of rare genetic variants in All of Us cohort patients with common variable immunodeficiency
Source: Front Genet. 2024 Oct 2;15:1409754. doi: 10.3389/fgene.2024.1409754 (PMC11479952; doi:10.3389/fgene.2024.1409754)
Supplement: Supplementary file 1 [file Table1.DOCX]

Supplementary Material

## Supplementary Figures

| *BLK* | *BLNK* | *BTK* | *CD19* | *CD20* | *CD21* | *CD27* | *CD70* | *CD81* | *CLEC16A* |
| --- | --- | --- | --- | --- | --- | --- | --- | --- | --- |
| *CR2* | *CTLA4* | *CXCR4* | *DCK1* | *DCLRE1C* | *DNMT3B* | *DOCK8* | *FCGR2A* | *FOXP3* | *ICOS* |
| *IGHM* | *IKZF1* | *IL12RB2* | *IL21* | *IL21R* | *IL21RB1* | *IRF2BP2* | *KMT2D* | *LONP1* | *LRBA* |
| *MLH1* | *MS4A1* | *MSH2* | *MSH5* | *NFKB1* | *NFKB2* | *ORC4L* | *PIK3CD* | *PIK3R1* | *PLCG2* |
| *PMS2* | *PRKCD* | *RAC2* | *RAD50* | *RAG1* | *RAG2* | *RFXANK* | *STAT3* | *TCF3* | *TINF2* |
| *TNFAIP3* | *TNFRSF13B* | *TNFRSF13C* | *TNFRSF7* | *TNFSF12* | *TTC37* | *TWEAK* | *UNC93B1* | *VAV1* | *XIAP* |
| *ZBTB24* |  |  |  |  |  |  |  |  |  |

**Supplemental Table 1.** List of putative CVID related genes based on current literature
